# Association of triglyceride/high-density lipoprotein cholesterol ratio with subclinical inflammation and 10-year cardiovascular disease risk as determined by Framingham risk scores in Korean male smokers

**DOI:** 10.17305/bb.2022.8135

**Published:** 2023-08-01

**Authors:** Myung Jae Seo, Jong-Koo Kim, Ji-Hye Kim, Tae-Ha Chung

**Affiliations:** 1 Department of Family Medicine, Wonju Severance Christian Hospital, Yonsei University Wonju College of Medicine, Wonju, Republic of Korea; 2 Research Group of Functional Medicine and Preclinical Disease, Yonsei University Wonju College of Medicine, Wonju, Republic of Korea; 3 Institute of Global Health Care and Development, Yonsei University Wonju College of Medicine, Wonju, Republic of Korea; 4 Department of Health Promotion, Severance Check-Up, Yonsei University Health System, Seoul, Republic of Korea

**Keywords:** Triglyceride/high-density lipoprotein cholesterol (TG/HDL ratio), insulin resistance, inflammation, cardiovascular disease (CVD).

## Abstract

Various risk factors and the multifactorial pathophysiology of cardiovascular disease (CVD) have been studied. We focused on the interaction between insulin resistance and inflammatory processes. Thus, we analyzed the association of triglyceride/high-density lipoprotein cholesterol (TG/HDL) ratio with subclinical inflammation and CVD risk in male smokers. We retrospectively reviewed medical records from the Health Promotion Center of Severance Hospital in Korea between 2015 and 2017. Subjects were currently smoking men aged 30–59, with leukocyte counts within the normal range, for a total of 1566. We grouped participants into four groups using the median TG/HDL ratio and the median leukocyte count. The odds ratio (OR) of high Framingham 10-year CVD risk (≥10%) was calculated using multiple logistic regression. The median Framingham 10-year CVD risk increased significantly from Group 1 (low leukocyte count, low TG/HDL ratio) to Group 4 (high leukocyte count, high TG/HDL ratio). The OR for Group 4 was 2.46 compared with Group 1 after adjusting for various CVD risk factors. In subgroup analysis, the OR of the highest leukocyte group was 1.57 compared to the lowest leukocyte group after adjusting for other variables. In conclusion, TG/HDL ratio and subclinical inflammation were positively related to CVD risk in Korean male smokers.

## Introduction

Cardiovascular disease (CVD) is the leading cause of mortality worldwide, and many risk factors of CVD have been reported. CVD risk factors include age, sex, diet, exercise, chronic stress, obesity, diabetes mellitus, hypertension, serum lipid levels, and sleep apnea [1–4]. Specifically, men face higher CVD and coronary heart disease mortality rates than women [5]. In addition, smoking is a significant risk factor for CVD regardless of age [6, 7]. Among various assessment tools for evaluating CVD risk, the Framingham risk score is commonly used to predict the CVD risk for 10 years. This scoring system identifies high risk for future CVD events and thus is an efficient decision-making method for clinicians and patients, contributing to appropriate patient education, preventive treatment, and lifestyle modification [8]. The pathophysiology of CVD is multifactorial and includes inflammation and insulin resistance. Chronic inflammatory pathways are involved in the pathogenesis of atherosclerosis and CVD, and studies have reported associations between increased inflammatory markers and diseases [9, 10]. Kim et al. [11] reported that total leukocyte count, a traditional inflammatory marker, was related to the risk of CVD. In addition, Festa et al. [12] suggested that chronic subclinical inflammation was associated with insulin resistance syndrome. Insulin resistance is major pathogenesis of diabetes mellitus, and its participation in hyperlipidemia and CVD has been studied [13]. Our previous study, which is a comparative study to find out which of triglyceride (TG) and TG/high-density lipoprotein cholesterol (TG/HDL) ratio showed a higher correlation with CVD, presented a positive relationship between TG/HDL ratio (a surrogate marker of insulin resistance) and Framingham 10-year CVD risk [14]. As an extension of our previous study, we focused on the interaction between TG/HDL ratio and inflammatory processes in CVD. In this aspect, smoking is significantly associated with inflammation in CVD [15]. Therefore, we select the population with current smoking status considered as a subject. Additionally, we excluded other conditions that could affect inflammatory markers and included only the male population, citing a study suggesting that self-reported smoking status in women is underestimated in Korea [16]. Finally, we combined TG/HDL ratio and within-normal-range leukocyte count to divide participants into four groups to assess associations with CVD risk in Korean male smokers.

## Materials and methods

We retrospectively reviewed the medical records of 26,176 participants aged 12 years or older who had a medical examination at the Health Promotion Center of Severance Hospital in Seoul, Republic of Korea, between 2015 and 2017. Participants who met one or more of the following criteria were excluded: missing anthropometric or questionnaire data; female sex; non or ex-smoker; aged < 30 or ≥ 60 years; current medication for dyslipidemia; positive result for hepatitis B antigens or hepatitis C antibodies test; leukocyte count < 4000 or > 10,000 cells/µL; high-sensitive C-reactive protein (CRP) ≥ 10 mg/L; history of cancer; history of heart, cerebral, thyroid, respiratory, renal, hepatobiliary, or rheumatologic disease; and failure to fast for 8 h before testing. After these exclusions (*n* ═ 24,610), we included 1566 participants aged 30–59.

Participants completed questionnaires about lifestyle and medical history, which included cigarette smoking, alcohol consumption, and physical activity characteristics. Current smoking status was defined based on the questionnaire. As the contents of alcohol intake included the frequency of consumption per week, we defined alcohol drinking as two or more times a week. Regular exercise was defined as moderate to vigorous exercise at least three times a week. Body mass and height were measured in units of 0.1 kg and 0.1 cm, respectively, with participants wearing light indoor clothes without shoes. Body mass index (BMI) was calculated by dividing weight (kg) by the square of height (m). Systolic blood pressure (SBP) and diastolic blood pressure (DBP) were measured on each patient’s right arm using a standard mercury sphygmomanometer (Kensei Industry Co., Ltd., Japan). Blood samples were obtained after an overnight fast of at least eight hours. Leukocyte counts were measured using the ADVIA 2120i Hematology System (Siemens Healthcare Diagnostic, Inc., NY, USA). Fasting plasma glucose, total cholesterol, TG, low-density lipoprotein cholesterol, high-density lipoprotein (HDL) cholesterol, and high-sensitive CRP levels were reported using the ADVIA 1800 Clinical Chemistry System (Siemens Healthcare Diagnostic, Inc., NY, USA). Hypertension was defined as follows: SBP ≥ 140 mm Hg, DBP ≥ 90 mm Hg, taking antihypertensive medication, or diagnosis by a physician. Diabetes was defined as the following: fasting glucose level ≥ 126 mg/dL, glycated hemoglobin ≥ 6.5%, taking antidiabetic medication, or diagnosis by a physician.

The Framingham risk score was used to predict the risk of severe cardiovascular events over the next ten years. This risk scoring system is based on the National Cholesterol Education Program Adult Treatment Panel III (NCEP-ATP III), and the score is calculated using six risk factors: sex, age, total cholesterol, HDL cholesterol, SBP considering hypertension treatment, and smoking status. The 10-year risk percentage is determined using the sum of all the calculated scores for each risk factor (Table S1) [17]. The estimated 10-year CVD risk is classified as low risk (<10%) or intermediate/high risk (≥10%). This study defined a high Framingham 10-year CVD risk as ≥ 10%.

**Figure 1.**
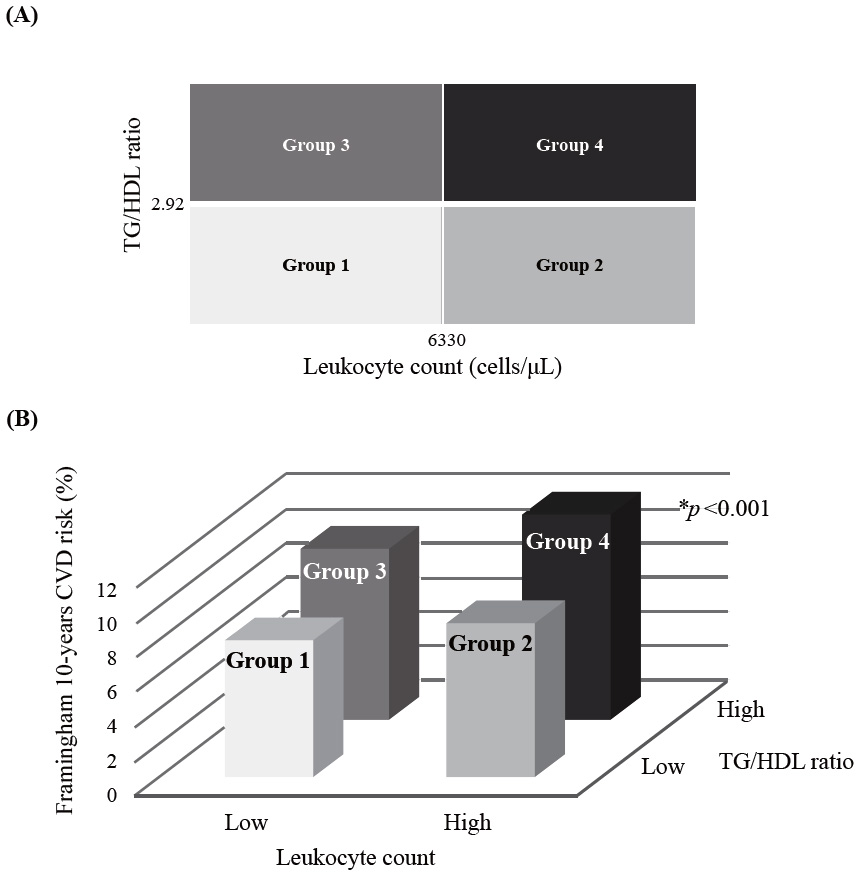
(A) Classification of four groups according to median values of leukocyte counts and TG/HDL ratio; (B) Median Framingham 10-year CVD risk (%) according to four groups. CVD: Cardiovascular disease; TG/HDL: Triglyceride/high-density lipoprotein cholesterol. *P value was obtained by using one-way analysis of variance.

In this study, the median TG/HDL ratio was 2.92, and the median leukocyte count was 6330 cells/µL after excluding participants with values, not within the normal range (4000–10,000 cells/µL). The study population was divided into two groups based on each median value, and these groups were combined to make a total of four groups. The schematic for the four groups (Group 1, Group 2, Group 3, and Group 4) is shown in Figure 1A. TG/HDL ratio with leukocyte count groups was categorized as follows: Group 1 (low TG/HDL ratio and low leukocyte count), Group 2 (low TG/HDL ratio and high leukocyte count), Group 3 (high TG/HDL ratio and low leukocyte count), and Group 4 (high TG/HDL ratio and high leukocyte count).

### Ethical statement

Our study was conducted in accordance with the ethical principles of the Declaration of Helsinki and was approved by the Institutional Review Board of Yonsei University Wonju College of Medicine (IRB No. CR321101).

### Statistical analysis

Distribution normality was assessed by determining skewness using the Kolmogorov–Smirnov test. General characteristics of the study population were analyzed among the four groups using a one-way analysis of variance or the Kruskal–Wallis test for continuous variables and the chi-square test for categorical variables. Continuous variables were presented as the mean with standard deviation or as the median with interquartile range, and categorical variables were presented as frequency. Additionally, we performed post hoc Tukey’s test on the data from a one-way analysis of variance. Odds ratio (OR) and 95% confidence intervals (CI) of high Framingham 10-year CVD risk were calculated for the four groups and subgroup analyses using multiple logistic regression analysis after adjusting for confounding factors. We used IBM SPSS Statistics for Windows, version 25 (IBM Corp., Armonk, NY, USA) for analysis. All statistical tests were two sided, and *P* values of < 0.05 were considered statistically significant.

## Results

Table 1 summarizes the clinical characteristics of the total population and four groups. The mean age was 45.7 years, and mean BMI, SBP, DBP, fasting plasma glucose, total cholesterol, and non-HDL cholesterol increased significantly from Group 1 to Group 4. The median TG/HDL ratio was significantly higher in the groups with higher leukocyte counts (Groups 2 and 4). Also, the groups with high TG/HDL ratios (Groups 3 and 4) had significantly higher median leukocyte values. Moreover, the prevalence of hypertension and diabetes mellitus and the median Framingham 10-year CVD risk was found to increase significantly from Group 1 to Group 4. Figure 1B shows the median Framingham 10-year CVD risk (%) according to the following results of four groups: 8 for Group 1, 9 for Group 2, 10 for Group 3, and 12 for Group 4.

**Table 1:** Clinical characteristics of the study population

	**Total**	**Group 1**	**Group 2**	**Group 3**	**Group 4**	***P* for trend^a^**	**Post hoc Tukeyʼs test (*P* value)**					
							**1 vs 2**	**1 vs 3**	**1 vs 4**	**2 vs 3**	**2 vs 4**	**3 vs 4**
*N*	1566	455	324	329	458							
Age (years)	45.7 (7.5)	45.5 (8.1)	45.6 (8.1)	45.6 (6.7)	46.0 (7.5)	0.662						
Body mass index (kg/m^2^)	25.0 (3.0)	24.0 (2.7)	24.3 (2.9)	25.5 (2.9)	26.0 (3.0)	<0.001	0.633	<0.001	<0.001	<0.001	<0.001	0.093
Systolic blood pressure (mmHg)	122 (13)	119 (12)	121 (13)	123 (13)	126 (12)	<0.001	0.034	<0.001	<0.001	0.414	<0.001	0.006
Diastolic blood pressure (mmHg)	81 (11)	78 (10)	80 (11)	82 (11)	84 (11)	<0.001	0.156	<0.001	<0.001	0.105	<0.001	0.007
Fasting plasma glucose (mg/dL)	103 (22)	98 (14)	100 (16)	104 (23)	108 (27)	<0.001	0.597	<0.001	<0.001	0.041	<0.001	0.034
Total cholesterol (mg/dL)	201 (34)	194 (32)	201 (34)	206 (34)	207 (35)	<0.001	0.846	<0.001	<0.001	<0.001	<0.001	<0.001
TG/HDL ratio	2.92 (1.88–4.69)	1.82 (1.37–2.26)	2.00 (1.51–2.42)	4.38 (3.57–5.96)	4.91 (3.80–6.73)	<0.001	0.846	<0.001	<0.001	<0.001	<0.001	<0.001
LDL cholesterol (mg/dL)	125 (33)	121 (32)	128 (33)	125 (31)	125 (34)	0.039	0.031	0.297	0.205	0.787	0.767	1.000
Non-HDL cholesterol (mg/dL)	153 (34)	140 (31)	146 (33)	162 (31)	163 (33)	<0.001	0.016	<0.001	<0.001	<0.001	<0.001	0.988
Leukocyte count (cells/µL)	6330 (5405–7330)	5270 (4795–5770)	7295 (6770–8195)	5540 (5040–5920)	7355 (6800–8300)	<0.001	<0.001	0.010	<0.001	<0.001	0.392	<0.001
C-reactive protein (mg/L)	0.4 (0.1–1.0)	0.2 (0.1–0.6)	0.5 (0.2–1.1)	0.4 (0.1–0.9)	0.6 (0.3–1.5)	<0.001	0.002	0.242	<0.001	0.403	0.331	0.004
Framingham 10-years CVD risk (%)	10 (6–16)	8 (4–12)	9 (5–12)	10 (8–16)	12 (8–16)	<0.001	0.489	<0.001	<0.001	<0.001	<0.001	0.479
Alcohol drinking (%)	75.0	74.5	74.4	73.3	77.3	0.584						
Regular exercise (%)	47.4	56.0	47.2	45.3	40.4	<0.001						
Hypertension (%)	29.7	20.7	24.4	31.9	40.8	<0.001						
Type 2 diabetes mellitus (%)	8.6	5.2	7.1	9.1	12.7	0.001						

Data are expressed as the mean (standard deviation), median (interquartile range), or percentage. Each group was categorized with TG/HDL ratio with leukocyte count groups: Group 1 (low TG/HDL ratio and low leukocyte count), Group 2 (low TG/HDL ratio and high leukocyte count), Group 3 (high TG/HDL ratio and low leukocyte count), and Group 4 (high TG/HDL ratio and high leukocyte count). CVD: Cardiovascular disease; TG: Triglyceride; HDL: High-density lipoprotein; TG/HDL: Triglyceride/high-density lipoprotein cholesterol; LDL: Low-density lipoprotein. ^a^P for trends were calculated using one-way analysis of variance test, Kruskal–Wallis test, or chi-square test.

Table 2 presents the results of multiple logistic regression analysis of the independent associations between the combination of TG/HDL ratio and leukocyte count and high Framingham 10-year CVD risk (≥10%). The OR (95% CI) of Group 4 for high Framingham 10-year CVD risk (≥10%) was 2.46 (1.81–3.35) compared to Group 1 after adjusting for BMI, fasting plasma glucose, low-density lipoprotein cholesterol, high-sensitive CRP, alcohol drinking, and regular exercise.

**Figure S1.**
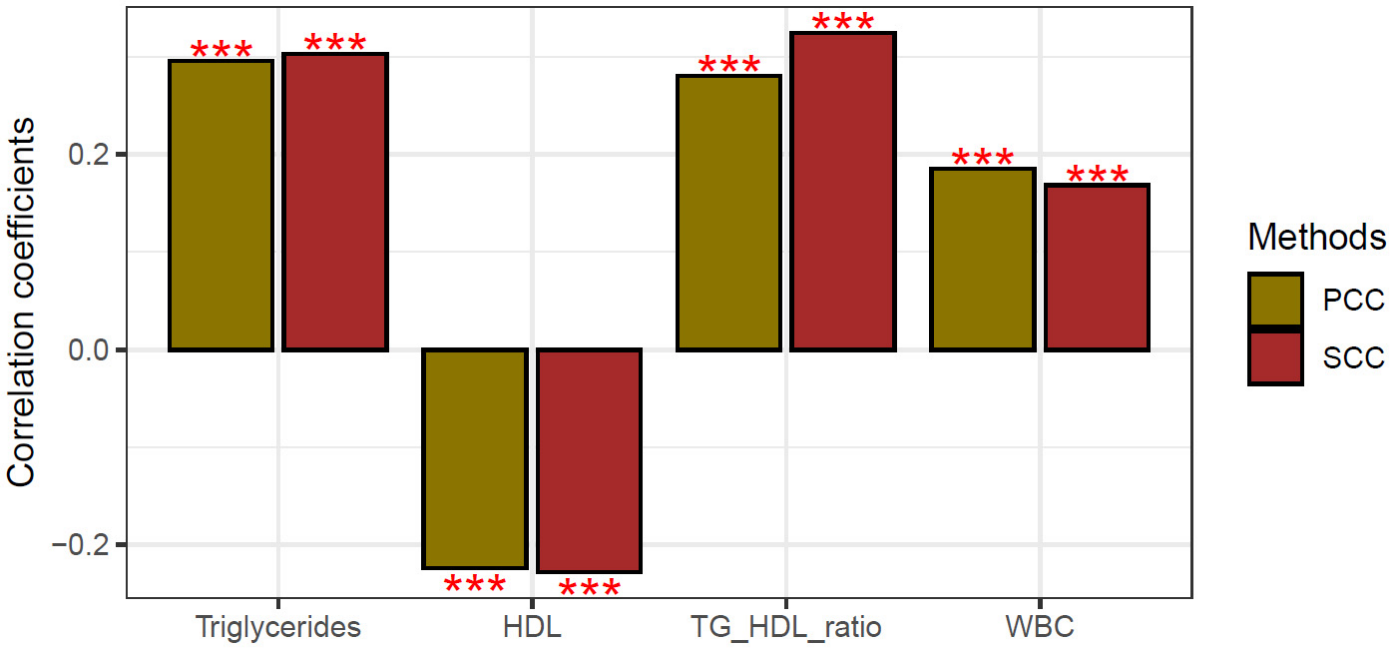
**Correlation analysis of TG, HDL cholesterol, TG/HDL ratio, WBC and Framingham 10-year CVD risk score in Group 3 and Group 4 (high TG/HDL ratio groups).** TG: Triglyceride; HDL: High-density lipoprotein; TG/HDL: Triglyceride/high-density lipoprotein cholesterol; WBC: White blood cell; CVD: Cardiovascular disease; SCC: Spearman correlation coefficient; PCC: Pearson correlation coefficient.

Table 3 describes the results of subgroup analysis by dividing the leukocyte count into quartiles for the 787 subjects in Groups 3 and 4 whose TG/HDL ratio was higher than the median value of 2.92. Clinical characteristics of the first to fourth quartiles were presented in Table S2. The OR (95% CI) of the highest leukocyte quartile for high Framingham 10-year CVD risk (≥10%) was 1.79 (1.12–2.84) compared to the lowest leukocyte quartile after adjusting for confounding factors. In addition, the correlation coefficients were shown in Table S3 and Figure S1 using correlation analysis of TG, HDL, TG/HDL ratio, leukocyte, and Framingham 10-year CVD risk score in Groups 3 and 4.

**Table 2:** Odds ratios and 95% confidence intervals for high Framingham 10-years CVD risk ≥ 10 % according to four groups

	**4 Groups**			
	**Group 1**	**Group 2**	**Group 3**	**Group 4**
Unadjusted	1.00 (reference)	1.19 (0.89–1.56)	2.21 (1.65–2.96)	2.62 (2.00–3.43)
Model 1	1.00 (reference)	0.97 (0.73–1.35)	1.96 (1.43–2.69)	2.25 (1.66–3.04)
Model 2	1.00 (reference)	1.03 (0.76–1.41)	2.10 (1.53–2.89)	2.46 (1.81–3.35)

Model 1: Adjusted for body mass index, fasting plasma glucose, LDL-cholesterol and C-reactive protein. Model 2: Adjusted for body mass index, fasting plasma glucose, LDL-cholesterol and C-reactive protein, alcohol drinking and regular exercise. CVD: Cardiovascular disease; LDL: Low-density lipoprotein.

**Table 3:** Odds ratios and 95% confidence intervals for high Framingham 10-years CVD risk ≥ 10 % according to leukocyte count quartiles in Groups 3 and 4 (high TG/HDL ratio groups)

	**Leukocyte count quartiles (cells/µL)**			
	**First quartile (4030–5840)**	**Second quartile (5841–6670)**	**Third quartile (6671–7670)**	**Fourth quartile (7671–9980)**
Unadjusted	1.00 (reference)	1.45 (0.95–1.96)	1.28 (0.83–1.96)	1.77 (1.15–2.74)
Model 1	1.00 (reference)	1.38 (0.89–2.14)	1.23 (0.79–1.91)	1.76 (1.11–2.78
Model 2	1.00 (reference)	1.37 (0.88–2.13)	1.24 (0.80–1.94)	1.79 (1.12–2.84)

Model 1: Adjusted for body mass index, fasting plasma glucose, LDL-cholesterol and C-reactive protein. Model 2: Adjusted for body mass index, fasting plasma glucose, LDL-cholesterol and C-reactive protein, alcohol drinking and regular exercise. CVD: Cardiovascular disease; LDL: Low-density lipoprotein.

## Discussion

Even though leukocyte counts were within the normal range, our study demonstrated that a combination of a high TG/HDL ratio and high leukocyte count was positively correlated with CVD risk. Previous studies demonstrated associations between CVD risk and TG/HDL ratio or inflammatory markers [11, 14]. However, this is the first study to compare CVD risk by combining these two indicators in male smokers with high CVD risk.

Previous studies reported that TG/HDL ratio was a surrogate marker of insulin resistance and was associated with metabolic syndrome, CVD, and CVD-related mortality [14, 18–21]. A large-scale study verified that those with TG/HDL ratio above the cut-off had a higher risk of insulin resistance, metabolic syndrome, and atherosclerosis severity than those without it [21]. Prasad et al. [19] presented a study of patients with non-obstructive coronary artery disease and found that patients who developed significant adverse cardiovascular events had higher TG/HDL ratios. A previous study described that a higher TG/HDL ratio was positively related to all-cause and CVD-related mortality in patients on peritoneal dialysis [20]. Leukocytes and inflammatory cytokines are involved in the processes of atherosclerosis and CVD [9–11]. Previous studies reported associations of various inflammatory markers, such as leukocytes, CRP, interleukin-6, plasminogen activator inhibitor-1, and fibrinogen with CVD [22–24]. In particular, the elevation of leukocyte count was significantly predictive of higher six-month mortality in patients with evidence of acute coronary syndrome [24]. In this study, leukocyte counts were classified into low (first quartile), intermediate (second and third quartiles), and high (fourth quartile) groups. Patients with high leukocyte counts had a six-month mortality risk of approximately eight-fold that of the lowest group. Margolis et al. [23] demonstrated that postmenopausal women with leukocyte counts in the highest quartile had approximately twice the risk of coronary heart disease death than women in the first quartile, independent of the serum level of CRP. Unlike previous studies, the strength of our study is that the concept of the TG/HDL ratio with subclinical inflammation can increase the risk of CVD in the future, even in people with a normal range of white blood cell counts.

Although the pathogenesis of CVD is complex and still not clearly understood, several mechanisms might explain the presence of a significant association between TG/HDL ratio with subclinical inflammation and increased risk of CVD in male smokers. Atherosclerosis is an established mechanism of CVD. The atherogenesis process induced by atherogenic diets that contain high levels of saturated fat has been described as the initiation point of inflammation [10]. When focusing on these inflammatory processes, leukocytes, mainly monocytes, adhere to endothelial cells and enter the intima, the inner layer of the artery [25]. Intimal monocytes accumulate lipid droplets, producing foam cell characteristics [26]. Foam cells secrete growth factors and cytokines that promote inflammation, leading to atherosclerotic lesions [27, 28]. Acute myocardial infarction usually originates from atherosclerotic plaque rupture [29]. Myocardial damage caused by myocardial ischemia increases cytokines, activates leukocytes, and recruits neutrophils [9]. Thus, previous studies revealed that inflammatory processes, including the activation of leukocytes, are organically involved in the development of atherosclerosis and CVD. Furthermore, insulin resistance and elevated TG levels affect inflammatory processes associated with atherosclerosis [30]. Insulin resistance stimulates adipocyte lysis and causes elevated serum-free fatty acids, increasing serum very low-density lipoprotein, apolipoprotein C-III, and TG [31]. Apolipoprotein B, which conveys TG and cholesterol to peripheral tissues, also increases, and subendothelial accumulation of apolipoprotein B is considered an initiating contributor to atherogenesis [32]. In addition, free fatty acids and apolipoprotein C-III activate Toll-like receptors and upregulate nuclear factor-kB adhesion molecules. This pathway modulates the inflammation involved in atherosclerosis [33, 34]. Even a study with a pediatric population analyzed TG/HDL ratio [35]. In this study, subjects with TG/HDL ratio ≥ 3.0 showed significant increases in the activity of cholesteryl ester transfer protein, a protein important for lipoprotein metabolism, and lipoprotein-associated phospholipase A2 activities, an enzyme considered a specific marker of vascular inflammation and CVD.

Various lifestyles associated with lipid imbalance or inflammation have been reported. Our results suggested that lifestyles related to high TG/HDL ratio and inflammation should be avoided to reduce CVD risks. A Chinese study reported the association of dietary patterns and physical activity level with the TG/HDL ratio [36]. In this study, researchers used the Food Frequency and Global Physical Activity Questionnaire to evaluate dietary and physical activity habits. They concluded that the active physical activity level, which means one or more hours of at least moderate-intensity activity daily, was related to a decrease in the risk of high TG/HDL ratio in both men and women. In addition, quartiles 2 and 3 of the dietary meat pattern and the highest quartile of the healthy dietary pattern were associated with lower risk of high TG/HDL ratio in men and women, respectively. Research on nutraceuticals demonstrated their effects on lipid disorders, involving the effects of soy milk and fish oil on TG and HDL cholesterol [37]. Furthermore, anti-inflammatory diets, representative of the Mediterranean or Nordic diets, were recommended as they included w-3 polyunsaturated fatty acids, phenolic compounds, and terpenes/terpenoids that are anti-inflammatory components [38]. Moreover, a previous study revealed that lower *n*−6/*n−*3 polyunsaturated fatty acids, higher vitamin D intake, and higher physical performance were associated with low-grade inflammation [39].

The strength of our study is that this is the first study to compare CVD risk by combining TG/HDL ratio and subclinical inflammatory markers. Furthermore, in groups with high TG/HDL ratio, we showed that leukocyte counts close to the upper limit of normal were associated with higher CVD risk. However, there are several limitations to consider when interpreting this study. First, although leukocyte count can fluctuate, it was measured only once. Thus, it was difficult to rule out the possibility of acute inflammation or other hematologic diseases. To overcome this limitation, we excluded participants with leukocyte counts <4000 or >10,000 cells/µL and high-sensitive CRP ≥ 10 mg/L. Second, as the data used questionnaires, subjects self-reported their medical history and lifestyle, including smoking, alcohol drinking, and exercise, and we should consider the possibility of overestimation or underestimation. In addition, smoking data was a categorical variable that could identify non-smokers, ex-smokers, and current smokers. Thus, the exact amount and frequency of smoking could not be determined. Further, the nutritional information which could affect lipid levels was not included in the questionnaires. Third, the data used in this study were a cross-sectional study. Therefore, causality should be excluded when explaining the results. Moreover, data were collected from a single center, and thus, generalizing the results is limited. Further, multicenter longitudinal studies with a large number of participants should be conducted.

## Conclusion

In conclusion, a combination of high-but-within-normal-range leukocyte count and high TG/HDL ratio was positively correlated with CVD risk in this study, especially in young or middle-aged male smokers. We identified that subclinical inflammation is a significant risk factor for CVD in Korean male smokers with a high TG/HDL ratio. Further studies using precise inflammatory biomarkers and various lipid indices need to confirm the relevance more clearly.

## Supplemental Data

**Table S1:** Estimate of 10-year cardiovascular disease risk for men

**Men**	
**Total points**	**10-year risk, %**
< 0	0
0–4	1
5–6	2
7	3
8	4
9	5
10	6
11	8
12	10
13	12
14	16
15	20
16	25
≥ 17	30

**Table S2:** Clinical characteristics of the subgroup population (Group 3 and Group 4)

	**Total**	**First quartile (4030–5840)**	**Second quartile (5841–6670)**	**Third quartile (6671–7670)**	**Fourth quartile (7671–9980)**	***P* value^a^**
*N*	747	168	197	187	195	
Age (years)	45.7 (7.0)	44.8 (6.6)	46.0 (7.0)	45.6 (7.1)	46.3 (7.0)	0.097
Body mass index (kg/m^2^)	25.8 (3.0)	25.2 (2.7)	25.8 (2.9)	26.0 (2.9)	26.2 (3.3)	0.003
Systolic blood pressure (mmHg)	124 (13)	123 (13)	123 (12)	126 (13)	126 (13)	0.011
Diastolic blood pressure (mmHg)	83 (11)	82 (11)	82 (11)	85 (11)	84 (10)	0.009
Fasting plasma glucose (mg/dL)	107 (26)	102 (21)	105 (22)	108 (27)	110 (30)	0.001
Total cholesterol (mg/dL)	206 (34)	204 (32)	206 (33)	207 (35)	208 (37)	0.277
TG/HDL ratio	4.76 (3.75–6.44)	4.23 (3.35–5.74)	4.64 (3.75–6.02)	4.87 (3.92–7.10)	5.20 (3.85–6.84)	<0.001
LDL cholesterol (mg/dL)	125 (32)	123 (27)	127 (31)	126 (34)	125 (35)	0.715
Leukocyte count (cells/µL)	6670 (5840–7670)	5175 (4722–5498)	6180 (5925–6400)	7020 (6830–7290)	8440 (8030–9280)	<0.001
C-reactive protein (mg/L)	0.5 (0.2–1.3)	0.3 (0.1–0.8)	0.5 (0.2–1.2)	0.5 (0.2–1.1)	0.8 (0.4–1.9)	<0.001
Framingham 10-years CVD risk (%)	12 (8–16)	10 (6–16)	12 (8–16)	12 (6–16)	12 (8–16)	0.020
Alcohol drinking (%)	75.1	67.9	74.1	79.1	78.5	0.054
Regular exercise (%)	41.1	44.0	44.7	38.5	37.4	0.356
Hypertension (%)	37.6	32.7	32.0	40.6	44.6	0.027
Type 2 diabetes mellitus (%)	11.0	5.0	9.8	10.4	17.9	0.001

Data are expressed as the mean (standard deviation), median (interquartile range), or percentage. CVD: Cardiovascular disease; LDL: Low-density lipoprotein; TG/HDL: Triglyceride/high-density lipoprotein cholesterol. ^a^*P* values were calculated using one-way analysis of variance test, Kruskal–Wallis test, or chi-square test.

**Table S3:** Correlation coefficients of TG, HDL cholesterol, TG/HDL ratio, WBC, and Framingham 10-year CVD risk score in Groups 3 and 4 (high TG/HDL ratio groups)

**Variable**	**PCC**		**SCC**	
	***R***	***P* value**	***R***	***P* value**
TG	0.296	<0.001	0.303	<0.001
HDL cholesterol	−0.224	<0.001	−0.228	<0.001
TG/HDL ratio	0.280	<0.001	0.324	<0.001
WBC	0.185	<0.001	0.169	<0.001

TG: Triglyceride; HDL: High-density lipoprotein; TG/HDL: Triglyceride/high-density lipoprotein cholesterol; WBC: White blood cell; CVD: Cardiovascular disease; SCC: Spearman correlation coefficient; PCC: Pearson correlation coefficient.
